# Beliefs about Obedience Levels in Studies Conducted within the Milgram Paradigm: Better than Average Effect and Comparisons of Typical Behaviors by Residents of Various Nations

**DOI:** 10.3389/fpsyg.2017.01632

**Published:** 2017-09-20

**Authors:** Tomasz Grzyb, Dariusz Dolinski

**Affiliations:** Faculty of Psychology in Wroclaw, SWPS University of Social Sciences and Humanities Warsaw, Poland

**Keywords:** obedience toward authority, better than average effect, social judgments, self, social comparisons

## Abstract

The article presents studies examining whether the better than average (BTA) effect appears in opinions regarding obedience of individuals participating in an experiment conducted in the Milgram paradigm. Participants are presented with a detailed description of the experiment, asked to declare at what moment an average participant would cease their participation in the study, and then asked to declare at what moment they themselves would quit the experiment. It turned out that the participants demonstrated a strong BTA effect. This effect also concerned those who had known the results of the Milgram experiment prior to the study. Interestingly, those individuals—in contrast to naive participants—judged that the average person would remain obedient for longer, but at the same time prior familiarity with the Milgram experiment did not impact convictions as to own obedience. By the same token, the BTA effect size was larger among those who had previously heard of the Milgram experiment than those who had not. Additionally, study participants were asked to estimate the behavior of the average resident of their country (Poland), as well as of average residents of several other European countries. It turned out that in participants’ judgment the average Pole would withdraw from the experiment quicker than the average Russian and average German, but later than average residents of France and England.

## Introduction

The series of experiments conducted by Milgram (1963, 1965) dedicated to the subject of obedience toward authority is among the most famous and most shocking in the history of social psychology. Demonstrating that the vast majority of people would follow the instruction to administer an electric shock of 450 V to another human being when told to do so by a university professor in the course of a supposed experiment on memory and learning came as a shock to not only the scientific community (Blass, 2004). Milgram (1974) also demonstrated that people presented with the plan of the aforementioned experiment and asked to predict the reactions of its participants commit a characteristic error. They assume that only a small percentage of people will agree to hit the last (30th) switch of the generator, and that the standard reaction of participants will be to refuse to carry out the experimenter’s instructions, thereby leading to a refusal to shock the alleged learner sitting in an adjacent room. This effect is a perfect example of a fundamental attribution error (Ross, 1977) consisting in overestimating the role of an individual’s dispositional traits, while at the same time failing to appreciate the effect of the situation that individual is operating in. People asked to predict the behavior of a Milgram experiment participant rather think about how evil and immoral a person would have to be to administer an electric shock to another person that could end his/her life, but not about what kind of situation could influence a normal person to engage in such behavior. However, Milgram convincingly showed that people do not appreciate the degree of obedience of the average person participating in his experiment. Thus, we can assume that they will be even more convinced that they would themselves not be persuaded by the experimenter to engage in behavior contrary to fundamental moral values.

In many studies where participants were asked to compare themselves to the average person, it turned out that the majority thought they were better—more physically attractive, more intelligent, healthier, more ethical (see Alicke and Govorun, 2005; Sedikides and Gregg, 2008 for review). The “better than average” (BTA) effect is particularly strong in respect of characteristics associated with morality (Allison et al., 1989; Van Lange and Sedikides, 1998). If we assume that people believe it is dispositive traits (and not situational factors) that play a primary role in determining the behavior of participants in Milgram’s studies, they should also assume that they possess better traits than the average person allowing them to oppose the experimenter’s pressure and to behave in accordance with morality and their own ethical standards. We thus posited the hypothesis that people should thus be convinced that they would conclude their participation in the Milgram experiment sooner than the average participant. In our study, we decided to check whether this really would be the case.

Because the Milgram experiments are quite well known not only within the psychological community but also among the public at large, it could be assumed that participants from diverse backgrounds would include both some who were unfamiliar with the Milgram studies, as well as others who had previously encountered descriptions of them. It is obvious that the latter should not err in estimating the obedience of an average person participating in the experiment (or at least the error should be smaller), but it seems an interesting question to ask whether those people will retain the conviction that they themselves would behave better than the average person and more quickly withdraw from the experiment. We thus hypothesize that people familiar with the results of the Milgram study will also modify their judgments of their own potential behaviors in that experiment (BTA effect is either smaller or entirely absent). We decided to see in our study if this really was the case.

The third question which we posed was one concerning the favoring of one’s own group in social comparisons (Festinger, 1954; Suls and Wills, 1991; Fisher, 2016). In other words, the question arises of whether people are convinced that an average representative of their own group during a Milgram experiment would demonstrate behavior more morally acceptable (i.e., refuse to press the next switch on the generator sooner) than an average representative of another group. Because participants in our study were Poles (and thus residents of Central Europe), we decided to ask them to predict the behavior of a typical Polish person as well as that of typical representatives of other nations from Europe. We selected nations which, during the period when the study was being conducted, were viewed by the majority in Poland in a negative light—Russians; rather neutrally–Germany; and quite positively—French and English (CBOS, 2016). In respect of the first category, the predictions are uniform (the average Pole, in the opinion of participants, will withdraw from the experiment sooner than the average Russian), whereas in the second a similar but weaker effect may be expected. In the third of the cases under consideration, the situation is somewhat more complicated. On the one hand, we may expect favoring one’s own group in such comparisons (a Pole should withdraw from the Milgram experiment sooner than an average Frenchman or Englishman), but on the other hand the positive stereotype held about people of those nationalities can mitigate this effect, or even make the average “other” perceived as refusing sooner than an average Pole to carry out the orders of the experimenter.

## Materials and Methods

The procedure was approved by the IRB (Komisja Etyczna ds. Badañ). The research was conducted with the assistance of *Ariadna*—the Polish research website (Polish counterpart of Amazon Mechanical Turk). There are approximately 100,000 respondents registered in the panel, aged 14–70, from among which a sample group was drawn. The panel is certified by the Polish Association of Public Opinion and Marketing Research Firms as well as the Quality Control Program of Pollsters’ Work, and operates in accordance with the international code ICC/ESOMAR. All the participants signed the informed consent form.

### Participants

There were 564 people randomly selected in more or less equal proportion in terms of sex (there were 268 women, constituting 52.5% of the sample). Sample size was determined before any data analysis. The youngest participant was 18 years old, and the oldest was 75. The mean age was 43.56 (*SD* = 15.65). In terms of place of residence, the participants were matched to the parameters of the general Polish population. Students and graduates of social science majors (sociology, psychology, and pedagogy) were excluded (that is to say, people who declared membership in that group concluded their participation in the study after completing the form, and their data was not retained). We did this with a view to the significant probability of some of them being familiar not only with the Milgram experiment itself, but also with the psychological mechanisms underlying the results he recorded. The study participants received points for participation in the study, which afterward they could exchange for various prizes.

### Procedure

The participants logged onto an internet portal and began completing a survey, starting with questions about sex, age, and place of residence. Next they were presented with a video roughly 6 min in length that detailed the procedure applied in the original experiment by Milgram. This was a presentation of slides containing pictures from the experiment, a description of the tasks given to the “teacher” and the “learner,” and a description of the actions taken by the experimenter together with a list of exhortations. In the course of the presentation, the recorded narrator’s voice gave details about the particulars of the experiment. At no time (neither in the presentation nor the voice-over) was information given about the results achieved.

After watching the presentation, the participants answered four control questions designed to verify how closely they had listened to the presented materials. If they responded correctly to at least three of four questions they were qualified to the next phase of the study.

During the next stage of the study they were asked the following question:

What do you think—at what moment did the average person (average from studies conducted around the world) cease participation in the experiment by refusing to press the next switch? Indicate the last switch that person pressed:

Participants were presented with a scale containing 30 switches, each of which were described exactly as they were in the Milgram experiment (voltage and label).

Participants responded on the same scale to questions regarding how they would behave in that experiment (Imagine that you yourself are participating in that experiment. Indicate the last switch you would press). The final element was a response to a question about the average value recorded in other countries (The experiment was conducted in countries around the world. Try to guess which was the last switch pressed by the average: Pole/German/Frenchman/Englishman). We adopted as our dependent variable the voltage of the last switch the participants thought they would be pressed (depending on the question: by them, by the average person, the average Pole, etc.). This means that, for example, the declaration of the 10th switch was considered to be a declaration of an electric shock with a strength of 150 V.

At the very end, the participants responded to a question about their previous familiarity with the Milgram experiment [Before today’s experiment were you familiar with the studies by Milgram, in which participants were encouraged to administer an electric shock to a “learner” (did you read about it or see a film)?]. The study then concluded, and the participants were thanked for completing the survey.

## Results

Because initial analyses demonstrated the absence of any effect for the sex of participants, this factor was not taken into consideration in further analyses.

To check whether assessments of predicted obedience among average representatives of various countries differ among themselves and whether this is associated with familiarity with the Milgram experiment, a mixed-design ANOVA was carried out. There was one within-subject factor (nationality of the residents) and one between-subject factor (familiarity with Milgram experiment). The results recorded for within-subject effect demonstrate a significant difference in assessments (sphericity not assumed, Huynh–Feldt correction applied): *F*(3.021) = 179.8; *p* < 0.001; Cohen’s *d* = 1.13. The between-subject effect was also significant: *F*(1,562) = 14.6; *p* < 0.001; Cohen’s *d* = 0.32. There was no significant interaction between effects. We have illustrated the differences by placing the averages of individual measures on a figure.

**FIGURE 1:**
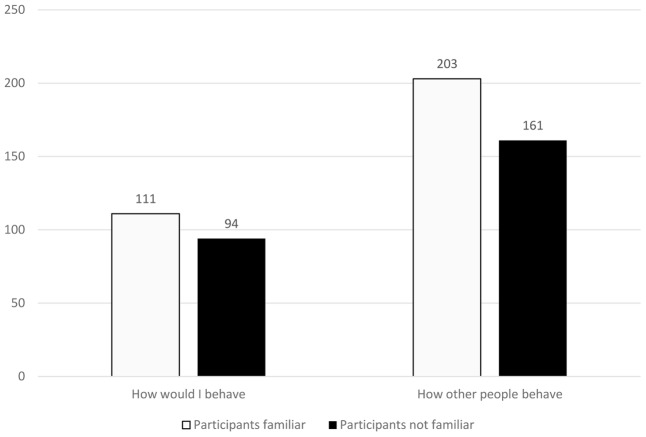
Means in questions “how would I behave” and “how other people behave” in group familiar and not familiar with Milgram experiment.

**FIGURE 2:**
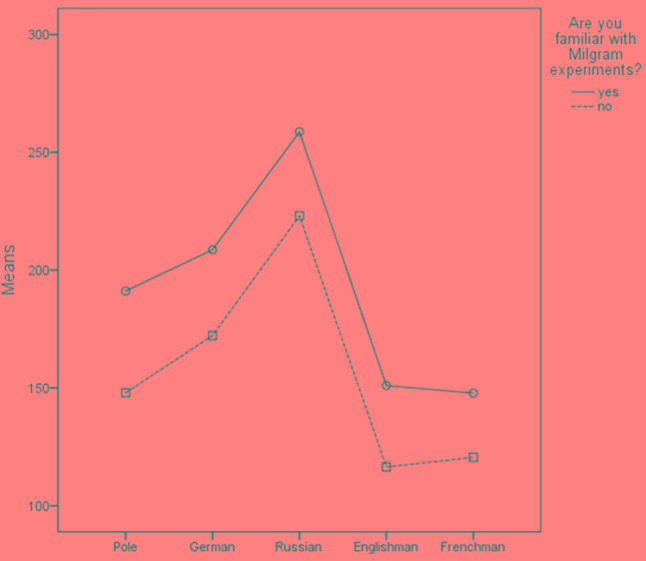
Means in questions on obedience among representatives of particular nations with consideration of prior knowledge of Milgram experiments.

We recorded a strong BTA effect. Participants felt that they would stop the experiment sooner than the average participant from Poland and average person around the world (see **Table 1**). Among all participants, 89 people admitted to previous familiarity with the Milgram experiment. They also demonstrated BTA effect (*M* = 110.56, *SD* = 97.59 for “I” vs. *M* = 202.92, *SD* = 122.18 for the average participant; *t* = 7.36, *p* < 0.001, Cohen’s *d* = 1.57). Characteristically, in their case the strength of the effect was even stronger than those unfamiliar with Milgram’s experiment (*M* = 93.88, *SD* = 93.73 for “I” vs. *M* = 160.64, *SD* = 105.19 for the average participant; *t* = 14.62, *p* < 0.001, Cohen’s *d* = 1.34; see **Figure 1**). The knowledge of participants about the results of Milgram’s experiment influenced their belief that the average person would proceed to press more switches on the generator, but did not influence their conviction as to their own behavior in such an experiment (there were no statistically significant differences in comparison to people who were unaware of the experiment—see **Figure 2**).

**Table 1:** The voltage of the last switch that the participants indicated in particular responses (myself, another, average Pole, etc.).

Nation	Mean	Standard error	95% Confidence interval	
			Lower bound	Upper bound
Average person	181.782	6.239	169.528	194.035
I (myself)	102.223	5.449	91.521	112.925
Pole	169.535	5.422	158.885	180.186
German	190.442	6.056	178.546	202.338
Russian	240.875	6.193	228.710	253.040
Englishman	133.737	4.755	124.398	143.076
Frenchman	134.189	4.980	124.408	143.970


**Table 1** contains the averages (with 95% confidence intervals) for assessments of obedience formulated by participants in reference to themselves and to an average person, Pole, Russian, German, etc.

As for social comparisons at the group level, the pattern of results was more complex (see **Table 2**). The participants were convinced that the average representative of their group would more quickly withdraw from the procedure than the average Russian and German, but later than the average Frenchman and Englishman.

**Table 2:** Estimates of the differences among particular averages—pairwise comparisons in mixed ANOVA model.

(I) Nation	(J) Nation	Mean difference (I-J)	Standard error	Significance	95% Confidence interval for difference	
					Lower bound	Upper bound
Average person	I (myself)	79.559	5.932	0.000	67.906	91.211
	Pole	12.246	4.919	0.013	2.585	21.908
	German	-8.660	5.984	0.148	-20.414	3.094
	Russian	-59.093	5.754	0.000	-70.396	-47.791
	Englishman	48.045	5.620	0.000	37.006	59.083
	Frenchman	47.593	5.750	0.000	36.298	58.888
I (myself)	Pole	-67.312	5.059	0.000	-77.249	-57.376
	German	-88.219	6.190	0.000	-100.377	-76.060
	Russian	-138.652	6.011	0.000	-150.459	-126.845
	Englishman	-31.514	5.197	0.000	-41.723	-21.306
	Frenchman	-31.966	5.309	0.000	-42.393	-21.538
Pole	German	-20.906	4.935	0.000	-30.599	-11.213
	Russian	-71.340	4.344	0.000	-79.871	-62.808
	Englishman	35.798	4.606	0.000	26.751	44.846
	Frenchman	35.347	4.969	0.000	25.587	45.107
German	Russian	-50.433	4.315	0.000	-58.909	-41.958
	Englishman	56.704	4.762	0.000	47.352	66.057
	Frenchman	56.253	4.757	0.000	46.909	65.597
Russian	Englishman	107.138	5.369	0.000	96.592	117.684
	Frenchman	106.686	5.601	0.000	95.684	117.688
Englishman	Frenchman	-0.452	2.886	0.876	-6.120	5.217

## Discussion

The results we achieved unambiguously point to the presence of the BTA effect. Participants felt that if they were to participate in such an experiment, they would be less obedient and pliable than the average participant. It is worth emphasizing that this effect was very strong and even gained in strength among individuals who were familiar with Milgram’s experiment.

The results we have recorded demonstrating that knowledge does not reduce bias in estimation of own vs. others obedience also gives rise to a more general question: would people’s knowledge of BTA modify the strength of that effect. Perhaps it is the case that, paradoxically, people who learn of the BTA effect and are then asked to estimate, for example, their sense of humor and that of the “average person” continue to feel that they themselves have an excellent sense of humor, while rating even lower the sense of humor of other people. Verification of this hypothesis would naturally require empirical study.

Results concerning social comparisons with average representatives of other nationalities turned out to be more complex. Favorizing one’s own group appeared only in conditions where the predicted behavior of a typical representative of the participant’s own group was compared with the predicted behavior of representatives from groups that Poles view either negatively or rather neutrally (CBOS, 2016). It should be observed here that in the case of convictions regarding obedience on the part of the average German, we may be facing associations with the obedience of Germans toward Hitler in the Nazi era. It is known that Milgram himself had the same associations when initiating his experiments. In respect of groups about which Poles have a very good opinion the effect of own-group favoring not only failed to appear, but in fact the opposite result was recorded. Participants thought that average representatives of those groups would behave better in the Milgram experiment (i.e., they would refuse to follow the experimenter’s commends sooner) than typical representatives of their own group.

Another interesting result would seem to be that familiarity with the Milgram experiment modifies the convictions of participants about the obedience of other people, but it does not change their conviction as to their own obedience. Psychological knowledge provides us with ample evidence that the motivation to defend one’s high estimation of oneself is both universal and strong (Greenwald et al., 1988; Pyszczynski et al., 2004). It would seem that the result under discussion here can also be interpreted through just such a lens. Participants do indeed take into consideration results recorded by Milgram in their thinking about the behavior of “people in general,” but they judge that they themselves as moral and reasonable people are not subject to these general truths, and they would behave differently—in a more appropriate manner consistent with social norms. We may consider to what extent the assessment of participants already familiar with the Milgram experimental procedure could be impacted by the generally negative opinion of it in the popular and popular science literature. For example, in 2015, “The Atlantic” described Milgram’s experiment as “One of Psychology’s Most Infamous Experiments” (Romm, 2015), and this is not an uncommon phrase to associate with Milgram’s work. Perhaps, then, the participants had an even greater motivation to present themselves in the context of the experiment as individuals resistant to the influence of the experimenter, remaining faithful to their moral principles.

From among the several results we have recorded, the most interesting, in our view, is the essentially zero-level impact of familiarity with the real results attained in the Milgram experiment on estimates of one’s own obedience. This is indeed an unusual result—it turns out that people aware of our real obedience in experiments are capable of using that knowledge when it concerns predicting the behavior of others. Yet they completely avoid applying that knowledge in reference to themselves—they estimate their obedience in a theoretical study as low when they are familiar with the Milgram experiment and its results, but also when they know nothing about them. Moore and Small (2007) point out that people generally have better (more precise) information about themselves (how frequently they lie, how frequently they solve a mathematical puzzle correctly) than about others. They must somehow infer the latter type of knowledge. The BTA effect demonstrated in the existing literature regards just these kinds of situations. Participants in our study familiar with the Milgram procedure were, from this perspective, in precisely the opposite situation: they know how an average person behaves in the experiment, but they are without definite information about themselves.

This result may also be exceptionally important from another perspective: after the publication of experiments conducted by Milgram (first in articles, later as a book), many people thought about how knowledge of them would impact social behaviors. In many educational programs (such as in secondary schools), Milgram is taught about during lessons as a way of demonstrating what a person under pressure is capable of doing. In light of our results, we may, however, fear that simple speaking about this will not change much in the behavior of pupils. Indeed, they will feel that “good people” can at times behave with cruelty. However, this will only refer to other people, not to the pupils themselves. This is a tremendous challenge, particularly from the practical perspective.

## Author Contributions

TG and DD were equally responsible for designing, conducting, and analyzing results of the research.

## Conflict of Interest Statement

The authors declare that the research was conducted in the absence of any commercial or financial relationships that could be construed as a potential conflict of interest.
